# Prediction of Chemotoxicity, Unplanned Hospitalizations and Early Death in Older Patients with Colorectal Cancer Treated with Chemotherapy

**DOI:** 10.3390/cancers14010127

**Published:** 2021-12-28

**Authors:** Jaime Feliu, Enrique Espinosa, Laura Basterretxea, Irene Paredero, Elisenda Llabrés, Beatriz Jiménez-Munárriz, Maite Antonio-Rebollo, Beatriz Losada, Alvaro Pinto, Ana Belén Custodio, María del Mar Muñoz, Jenifer Gómez-Mediavilla, María-Dolores Torregrosa, Gema Soler, Patricia Cruz, Oliver Higuera, María-José Molina-Garrido

**Affiliations:** 1 Oncology Department, La Paz University Hospital, IDIPAZ, CIBERONC, UAM-AMGEN Cathedra, 28046 Madrid, Spain; eespinosa@salud.madrid.org (E.E.); alvaro.pinto@salud.madrid.org (A.P.); ana.custodio@salud.madrid.org (A.B.C.); patricia.cruz@salud.madrid.org (P.C.); oliver.higuera@salud.madrid.org (O.H.); 2 Oncology Department, Donostia University Hospital, 20014 Donostia, Spain; laura.basterrecheabadiola@osakidetza.eus (L.B.); jgomez@onkologikoa.org (J.G.-M.); 3 Oncology Department, Doctor Peset University Hospital, 46017 Valencia, Spain; irene.paredero@hospitalprovincial.es (I.P.); torregrosa_dol@gva.es (M.-D.T.); 4 Oncology Department, Insular University Hospital of Gran Canarias, 35016 Las Palmas, Spain; ellaval@gobiernodecanarias.org; 5 Oncology Department, Centro Integral Oncológico Clara Campal, 28050 Madrid, Spain; bjimenez@hmhospitales.com; 6 Oncohematogeriatrics Unit, Institut Català d’Oncologia, IDIBELL Hospitalet, 08908 Barcelona, Spain; marebollo@iconcologia.net (M.A.-R.); gsoler@iconcologia.net (G.S.); 7 Oncology Department, University Hospital of Fuenlabrada, 28942 Madrid, Spain; beatriz.losada@salud.madrid.org; 8 Oncology Department, Hospital Virgen de la Luz, 16002 Cuenca, Spain; madelms@sescam.jccm.es (M.d.M.M.); mjmolinag@sescam.jccm.es (M.-J.M.-G.)

**Keywords:** cancer, older, frailty, geriatric assessment, chemo-toxicities, unplanned hospitalizations, prognostic, early death

## Abstract

**Simple Summary:**

Chemotoxicity, unplanned hospitalizations (Uhs) and early death (ED) are common among older patients with cancer who receive chemotherapy. Our objective was to determine factors predicting these complications. A predictive score for these three complications based on geriatric, tumor and laboratory variables was developed in a series of 215 older patients with colorectal carcinoma receiving chemotherapy. The use of this score may reliably identify patients at risk to have excessive toxicity with chemotherapy, UH or ED, thus helping to plan treatment, implement adaptive measures, and intensify follow-up.

**Abstract:**

Purpose: To identify risk factors for toxicity, unplanned hospitalization (UH) and early death (ED) in older patients with colorectal carcinoma (CRC) initiating chemotherapy. Methods: 215 patients over 70 years were prospectively included. Geriatric assessment was performed before treatment, and tumor and treatment variables were collected. The association between these factors and grade 3–5 toxicity, UH and ED (<6 months) was examined by using multivariable logistic regression. Score points were assigned to each risk factor. Results: During the first 6 months of treatment, 33% of patients developed grade 3–5 toxicity, 31% had UH and 23% died. Risk factors were, for toxicity, instrumental activities of daily living, creatinine clearance, weight loss and MAX2 index; for UH, Charlson Comorbidity Score, creatinine clearance, weight loss, serum albumin, and metastatic disease; and for ED, basic activities in daily living, weight loss, metastatic disease, and hemoglobin levels. Predictive scores were built with these variables. The areas under receiver operation characteristic (ROC) curves for toxicity, UH and ED were 0.70 (95% CI: 0.64–0.766), 0.726 (95% IC: 0.661–0.799) and 0.74 (95% IC: 0.678–0.809), respectively. Conclusion: Simple scores based on geriatric, tumor and laboratory characteristics predict severe toxicity, UH and ED, and may help in treatment planning.

## 1. Introduction

Colorectal carcinoma (CRC) is commonly diagnosed in older patients, with almost 50% of patients being ≥70 years of age [1,2]. The incidence will grow in coming years due to progressive ageing of the population.

Overall health condition, functional dependence grade, physical functional reserve, comorbidities, and geriatric conditions show wide variation among the older population Ageing brings physiological changes that may modify the pharmacokinetics and pharmacodynamics of the drugs as well as the tissue’s tolerance, leading to a narrowing of the therapeutic margin and an increased toxicity [3]. Considering this and the low inclusion rate of older people in clinical trials [4,5], their treatment poses a challenge.

Chemotherapy can improve survival and relieve symptoms in older patients with cancer [6,7], but may also produce serious toxicities and derange preexisting conditions [3]. Such complications can lead to hospitalization, which has a significant impact on the patient’s quality of life, causing a decline in functional capacity and loss of independence, and leading to institutionalization in many cases [8,9]. It follows that planning chemotherapy should balance risks and benefits [10].

Standard oncology tools do not properly identify older patients at higher risk of developing chemotherapy-related complications [10]. Geriatric assessment (GA) is a multidimensional tool that evaluates patient’s daily life and health status. GA may help clinicians to predict poor treatment outcomes as toxicity, morbidity, and mortality [11]. However, GA also requires multidisciplinary specialist knowledge, facilities and time [11,12]. For this reason, simpler and more practical predictive tools may help in the process of decision making.

A limited number of studies have evaluated GA to predict the toxicity of chemotherapy in patients with CRC [12,13,14]. A limited predictive ability of geriatric screening tools in relation with chemotoxicity, functional decline and survival in CRC has been reported [15,16,17]. In addition, the studies that developed the two main predictive scores for toxicity of chemotherapy in older patients included a limited number of gastrointestinal malignancies, 27% [18] and 12% [19], respectively. 

Our objective was to identify factors predicting grade 3–4 toxicity, unplanned hospitalization (UH) and early death (ED) (death within the first six months) in older patients with colorectal cancer initiating chemotherapy. Estimating the risk of 6-month mortality is relevant regardless of the disease stage. In the case of localized tumors, it would be useful to identify those older adults at risk of early mortality; thus, long term gains in survival with adjuvant therapy may not be achieved. In advanced disease, a reliable prognostic estimation would allow: (1) to adapt therapy to life expectancy; (2) to provide accurate information to patients; (3) to optimize medical and social resources; (4) to group patients with similar prognosis for clinical research.

All these relevant outcomes could be useful to plan therapeutic strategies in accordance with life expectancy and toxicities while avoiding unnecessary treatments and toxicities.

## 2. Materials and Methods

This prospective and multicenter study included 215 patients from February 2014 to June 2018 at the following departments of oncology in Spain: La Paz University Hospital, Donostia University Hospital, Dr. Peset University Hospital. Insular de Gran Canarias University Hospital, Centro Integral Oncológico Clara Campal, Institut Català d’Oncologia, Fuenlabrada University Hospital and Virgen de la Luz Hospital. Inclusion criteria were: (1) age ≥ 70 years, (2) histological or cytological confirmation of CRC cancer in any stage, (3) Eastern Cooperative Oncology Group performance status (PS) (ECOG-PS) 0–2, (4) initiation of adjuvant or first-line metastatic chemotherapy, and (5) the ability to read Spanish (questionnaires for geriatric assessment were in Spanish). All patients completed the informed consent form. The study was approved by the institutional review board at each participating center. The names of the Ethics Committees that approved the study, along with the number/ID of the approvals, are: Comité Ético del H. Universitario del H. La Paz, 13 June 2013 (IRB number: 1349). Comité Ético del H. Universitario del Hospital OSI Bilbao Basurto, 17 July 2014 (IRB number: 0318). Hospital Universitario Dr. Peset, 15 May 2016 (IRB number: 0031/6). Comité Ético del H. Universitario del Complejo Hospitalario Universitario Insular-Materno Infantil, 7 April 2017 (IRB number: 9161). Comité Ético del H. Universitario del Hospital Universitario de Fuenlabrada 5, December 2016, (IRB number: 10123). Comité Ético del H. Universitario de Bellevitge 17 July 2013 (IRB number: 0028/13). Comité Ético del Hospital Virgen de La Luz Hospital, 8 July 2013 (IRB number: 1712).

### 2.1. Study Scheme

Full clinical staging was performed according to routine clinical practice. Before starting chemotherapy, patients completed a baseline geriatric assessment (GA) (Supplementary Table S1). The questionnaire was delivered by a research nurse; one part was completed by the patient and another one by the health professional. The latter included the following items: ECOG PS [20], comorbidities (assessed by the Cumulative Illness Rating Scale Geriatrics (CIRS-G) and Charlson index) [21,22], frailty by the short physical performance battery (SPPB) (comprising the 4 m gait speed, standing balance, and five-repetition chair-stand test) [23,24], body mass index (BMI), percentage of weight loss in the last 6 months and the cognitive status by the Short Portable Mental Status Questionnaire (Pfeiffer´s test) [25], which assigns a score from 0 to 10 mistakes. The patient reported measures of: functional status (basic activities in daily living (ADL) [26], instrumental activities in daily living (IADL) by the Lawton Index [27], number of falls in the last six months, medications, nutrition, psychological state [28], social support and function [29,30], ability to take medications unassisted, and the Vulnerable Elders Survey-13 [31]. A member of the health care team assisted those who needed help with completing the questionnaires. The clinical variables collected were age, gender, education, marital status, household composition, hearing, cancer subtype and stage, and selected blood tests obtained before treatment: hemoglobin (normal ≥ 12 g/dL), white blood cell count (normal 3700–11,600 × 10^3^ μL), platelets (normal 125–350 × 10^3^ μL), basal creatinine (normal 0.8–1.3 mg/dL), albumin (normal 35–52 g/dL), aspartate aminotransferase (normal < 35 units/L), alanine aminotransferase (normal < 45 units/L), gamma glutamyl transferase, (normal < 55 units/L) alkaline phosphatase (normal 30–120 units/L), and creatinine clearance (normal ≥ 60 mL/min) [32]. All-cause mortality was captured from the hospital database and national death registry.

The risk of chemotherapy-induced toxicity for every chemotherapy regimen administered was estimated with the MAX2 index toxicity [33]. The MAX2 index is a standardized tool that summarizes the overall risk of severe chemotherapy-induced toxicity based on an average study using data from published clinical trials. Briefly, the MAX2 index is the average of the highest frequency of both grade 4 hematologic toxicity and grades 3–4 non-hematologic toxicity, with higher scores indicating higher risk of toxicity. It is reproducible across cancer types and studies and is sensitive to toxicity differences among chemotherapy regimens. 

Standard therapy was defined as combination chemotherapy or monotherapy at standard doses according to guidelines. Reduced therapy was defined as either (1) combination or single-agent chemotherapy at reduced doses or (2) single-agent chemotherapy at standard dose when combination chemotherapy was the first option according to guidelines [34]. The use of reduced doses was decided by the treating oncologist based on patients’ frailty.

All patients were followed up for at least 6 months or until death. Unscheduled visits and emergency department admissions were also collected. Toxicity was assessed using the Common Terminology Criteria for Adverse Events (CTCAE) v.4.03 [35]. UH and deaths occurring in the 6 months following the start of treatment were recorded. UH was defined as any inpatient admission to an acute care hospital that began after the day of starting chemotherapy and that could not be foreseen. It could happen either on a non-emergency or an emergency basis [36].

### 2.2. Statistical Analysis

Descriptive statistics characterizing patient groups were provided. The X^2^ test was used to examine the association between categorical variables and independent t tests for continuous variables. We performed a correlation assessment using the Spearman’s rho test as appropriate for categorical variables. Multicollinearity between variables was defined as a rho test value ≥0.50. An evaluation of predictors was performed by using logistic regression. Univariate models were first fitted for all prognostic factors. Significant variables at the 5% level were selected for inclusion in the multivariable model. Odds ratios (Ors) were reported with their 95% Cis. *p* < 0.05 was considered statistically significant for all comparisons. Interactions between selected factors (age, gender or tumor stage) were evaluated introducing interaction terms to the model, one at the time, in the multivariate logistic regression. However, no significant interaction was found between risk factors included in different models. The optimal cut-point for the continuous variables was determined using the Youden index, and the categorical variables were dichotomized according to clinically relevant cutoffs. The amount of accounted variance was determinate with the Nagelkerke correlation coefficient (R2). Model calibration and discrimination were assessed by the Hosmer–Lameshow test and the area under the receiver operating characteristic (ROC) curve [37,38]. 

Each factor was assigned a particular score based on its β coefficient to develop prognostic scores. The β coefficient for each risk factor was divided by the lowest β coefficient and rounded to the nearest whole number [39,40]. The risk score was then applied to each patient. The sample was divided into three risk strata (low, medium, and high risk) on the basis of approximate tertiles of risk score. We compared the risk groups by chi square testing. We used the bootstrap method (1000 repetitions) for internal validation of the risk score. Bootstrap validation is a method of random resampling from a given set of samples to simulate the effect of drawing samples from the same population. Analyses were carried out by with SPSS software (version 18; SPSS, Chicago, IL, USA).

## 3. Results

### 3.1. Patients’ Characteristics

Two hundred twenty patients completed the baseline assessment. Two of them withdrew consent early and three moved to another center after the first cycle of treatment, and were excluded from the analysis; thus, the series finally included 215 patients. 

Baseline patient characteristics, including demographics, GA, chemotherapy, and laboratory findings, are shown in Table 1. Median age was 78 years (range 70–92) and 40% had ≥80 years; most patients had a good performance status with Eastern Cooperative Oncology Group (ECOG) ≤1 (94%), staging was I–III (51%) and IV (49%). Chemotherapy was administered in the adjuvant or neoadjuvant setting in 51% of patients and as first line in 49%. Fifty-six percent of patients received combination chemotherapy. Chemotherapy was delivered at standard doses in 47% of patients, more commonly in those aged 70–79 years (54% vs. 38%; *p* < 0.05). Primary prophylaxis with granulocyte colony-stimulating growth factors was used in 8% of patients. 

### 3.2. Geriatric Assessment

Fourteen percent of patients had at least three errors in the Pfeifer test, which denotes cognitive impairment. A thorough study of cognitive function considering cultural and study levels, confirmed this impairment in 12% of them. Ten percent of patients required assistance from the health care team (explaining the meaning of some questions). Only 4% of patients required that a relative completed the questionnaire.

One third of patients had two or more comorbid conditions according Charlson index. Fifty-five percent and 16% of patients had IADL and ADL disabilities, respectively (Table 1). Fifteen percent had had at least one fall in the last 6 months. The SPPB score was ≤6 also in 14% of patients. Fourteen percent had ≥3 errors in the Pfeiffer test. There was unintentional weight loss >5% in 34% and ≥10% in 12% of patients. Fifty-two percent of patients had a VES 13 score >2, which indicates fragility, being more common among patients older than 80 (73% vs. 39%; *p* < 0.0001).

### 3.3. Chemotherapy Toxicities, Unplanned Hospitalizations and Death

After a follow-up of 6 months, 34% of patients had G3-5 toxicity. Hematologic and nonhematologic grade 3–5 sides effects occurred in 17% and 28% of patients, respectively. The most common grade 3–5 hematologic toxicities were neutropenia (8%) and anemia (6%). The most common grade 3–5 non hematologic toxicities were fatigue (12%), diarrhea (10%), and neuropathy (6%). Two patients died as a result of chemotherapy toxicity (febrile neutropenia and sepsis, and diarrhea). Twenty-eight percent of patients had an UH, most commonly due to infection (11%), cancer progression (10%), toxicity (5%) and falls (2%). Twenty-two percent of patients died in the six months following the initiation of therapy. The main causes of death were disease progression (10%), comorbidities (9%) and toxicity (1%).

### 3.4. Predictive Variables Associated with Grade 3–5 Toxicity, Unplanned Hospitalizations and Death

Univariate analysis was performed to analyze domains of GA, clinical, and laboratory parameters (Table 2). Factors related to the development of grade 3–5 toxicity were IADL ≤ 7, creatinine clearance ≤ 50 mL/min, weight loss ≥ 5%, and a MAX2 index ≥ 0.45. Factors related to unplanned hospitalizations were ECOG PS 2, IADL ≤ 7, ADL ≤ 5, Charlson Comorbidity Score ≥ 2, creatinine clearance ≤ 50 mL/min, albumin ≤ 35 g/dL, weight loss ≥ 5% and metastatic disease. Risk factors for early death were ADL ≤ 5, Charlson Comorbidity Score ≥ 2, weight loss ≥ 5%, metastatic disease, serum albumin levels ≤ 35 g/dL, and hemoglobin levels < 11 g/dL.

In multivariate logistic regression, variables associated with grade 3–5 toxicity were IADL, creatinine clearance, weight loss and MAX2 index (Table 3). Independent variables for UH were disease stage, weight loss, creatinine clearance, albumin and Charlson comorbidity score (Table 4). Variables related to ED were disease stage, weight loss, ADL and hemoglobin (Table 5).

### 3.5. Predictive Model for Chemotherapy Grade 3–5 Toxicity, Unplanned Hospitalizations and Death

**Figure 1:**
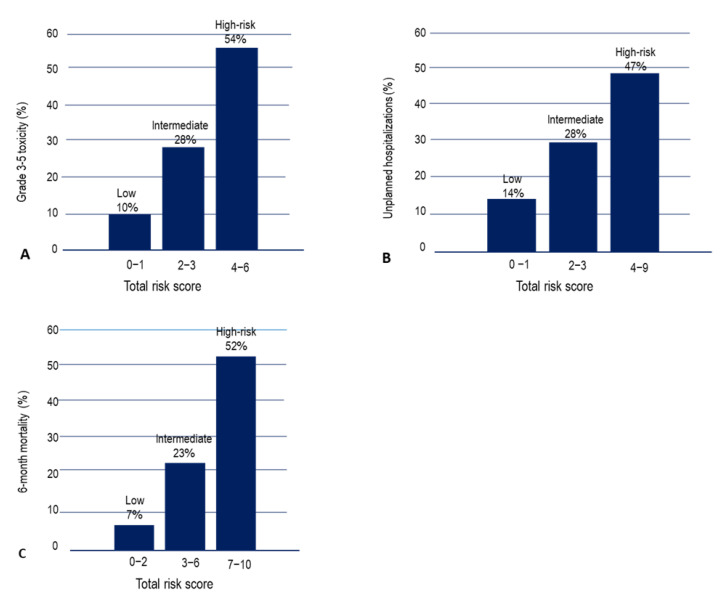
Ability of different risk score to predict grade 3–5 toxicity (**A**), unplanned hospitalizations in 6 months (**B**) and 6-month mortality (**C**).

Variables identified in the multivariate analysis were assigned a value depending on their β coefficient. The values were used to generate a predictive score. The risk score was applied to each patient, and patients were classified into three categories on the basis of the risk of toxicity: low risk (0–1 points: 10% toxicity 3–5), intermediate risk (2–3 points: 28% grade 3–5 toxicity), and high risk (4–6 points: 54% grade 3–5 toxicity) (Figure 1). The proportion of patients classified as low, intermediate, or high risk were 16%, 57%, and 27%, respectively. There was a significant difference in the grade 3–5 toxicity among the risk groups (*p* < 0.001). The area under receiver operation characteristic (ROC) curve was 0.70 (95% CI: 0.64–0.766) (Supplementary Figure S1). 

A different predictive score for UH was developed. Patients were classified into 3 categories: low risk (0–1 points: 14% 6-month UH rate), intermediate risk (2–3 points: 28% 6-month UH rate), and high risk (4–9 points: 47% 6-month UH rate) (Figure 1). The proportion of patients classified as low, intermediate, or high risk were 21%, 38%, and 41%, respectively. There was a significant difference in the UH rate among the risk groups (*p* < 0.001). The AUC was 0.726 (95% IC: 0.661–0.799) (Supplementary Figure S1). Exploratory analyses were performed to calculate the ROC of the model by using the total risk score according to stage: localized (0.711) and disseminated (0.739).

Finally, another score predicted 6-month ED and classified patients as low risk (0–1 points: 7% 6-month mortality rate), intermediate risk (3–6 points: 23% 6-month mortality rate), and high risk (7–10 points: 52% 6-month mortality rate). The proportion of patients classified as low, intermediate, or high risk were 42%, 38%, and 20%, respectively (Figure 1). There was a significant difference in the 6-month mortality rate among the risk groups (*p* < 0.001). The AUC was 0.74 (95% IC: 0.678–0.809) (Supplementary Figure S1). Exploratory analyses were performed to calculate the ROC of the model by using the total risk score according to stage: localized (0.771) and disseminated (0.723).

Calibration of the three final models was assessed using the Hosmer–Lemeshow goodness of fit test. *p* values of 0.58 (95% CI, 0.53 to 0.64) for toxicity, 0.296 (95% CI, 0.23 to 0.34) for UH and 0.562 (95% CI, 0.57 to 0.67) for 6-month mortality suggest that the models are accurate.

## 4. Discussion

Treatment planning in older patients with cancer should consider the risks of developing serious toxicity, UH and ED. Our results in patients with CRC aged 70 or older identified IADL, creatinine clearance, weight loss and the MAX2 index as risk factors for grade 3–4 toxicity; clinical stage, weight loss, creatinine clearance, albumin and Charlson comorbidity score for UH; and clinical stage, weight loss, ADL and hemoglobin for ED. The combination of geriatric, tumor and laboratory variables was used to develop accurate scores for the three end points.

Models to predict toxicity of chemotherapy have been developed for the general population of older patients with cancer [18,19,41]; there is also experience in the specific area of CRC [13,14]. Dependence on IADL was associated with early discontinuation of chemotherapy in older patients with lung cancer [42] and with 3–4 toxicities in older patients treated for metastatic colorectal cancer with first-line chemotherapy [14]. Likewise, IADL has been identified as significant in two other studies including a variety of tumor types [18,19], which suggests that the patient’s functional performance is relevant in this regard. Grip strength and ECOG PS, which also relate to functional performance, have also been found as significant in patients with CRC [13]. Renal function appeared as significant in the present study and in other studies [18,43,44]. Renal function deteriorates with age and chemotherapy-related toxicity increases by 12% for every 10 mL/min decrease in creatinine clearance [43]. Nutritional status has also been related with toxicity [13,19,41]. We decided to use the percentage of weight loss to estimate this parameter, although other methods have been proposed: mini-nutritional assessment (MNA) [19], serum protein levels [41] or serum albumin levels [13]. As expected, treatment aggressiveness correlated with toxicity. The risk of developing grade 3–4 toxicity has been previously correlated with standard-dose combination chemotherapy [13,18,41]. We and others [19] used the MAX2 index, which summarizes the overall risk of severe chemotherapy-induced toxicity based on an average study using data from published clinical trials [33]. This index is part of the Chemotherapy Risk Assessment Scale for High-Age Patients (CRASH score) [19]. In our series this index performed better than single parameters such as “combined chemotherapy” or “dose of chemotherapy”. It was striking that the chemotherapy regimen (single agent vs. combination) did not predict toxicity. The most likely reason, as previously stated by other authors [17], is that older patients usually received reduced doses. One study reported that cognitive impairment in physically independent patients with CCR living at home can cause difficulty in taking medication or using the telephone and be responsible for toxicities [14] We did not find such limitations, probably due to the low number of patients with cognitive impairment in our series.

Hospitalization negatively impacts quality of life and may lead to an irreversible decline in functional capacity and loss of independence in the elderly. It also increases health costs [45]. The identification of risk factors for UH could lead to the implementation of preventive measures. Risk factors for UH have been identified in the general population of patients with cancer [46,47,48] and older patients with cancer [49,50]. A predictive score for UH in old patients receiving chemotherapy has also been proposed [51]. Thirty-one percent of our patients had an UH in the 6 months following the start of chemotherapy. Variables related to the tumor (disease stage), pharmacokinetics (renal function and albumin), nutritional status (weight loss and albumin) and comorbidities (Charlson comorbidity score) correlated with UH. Other studies have also found that comorbidity [46,47,48,49], poor nutritional status [50], low albumin level [46,51], cognitive function [14] and depression [14] increase the risk of UH. Serum albumin decreases 15–20% with age, even more in the case of poor nutrition. This may result in an increase in the free fraction of the drug in plasma, as described for cisplatin, etoposide, taxanes, or methotrexate [52], thus increasing adverse events. However, only 5% of UH in our series were due to toxicity, which suggests that the association with albumin and weight loss rather indicates frailty or sarcopenia. These two geriatric syndromes increase the risk of hospitalization [53,54].

One-fourth of our patients died in the first six months after starting treatment. Prediction of ED would preclude the use of any adjuvant therapy in non-metastatic tumors, as the long-term gains in survival with this therapy may not be achieved. In patients with metastatic disease, this information would have allowed to make better decisions regarding life expectancy and priorities, also helping in treatment planning and resource optimization. Some scores and nomograms estimate the risk of death at 1, 2, 3 and 4 years, both for the elderly population as a whole [55,56,57] and for older patients with cancer [58,59]. These tools could help to decide about the initiation of adjuvant therapy. Prognostic factors have been identified and predictive scores developed to predict ED in older patients, whether they receive chemotherapy [60,61] or not [62,63,64]. These studies, as well as ours, point out to tumor stage, nutritional status and performance status as key factors in this regard [60,61,62,63,64]. Similar results have been reported in two other studies in gastrointestinal tumors [12,13]. Anemia has also been correlated with ED. Although there is not a clear explanation for such a correlation, cancer leads to a pro-inflammatory stage that may inhibit hematopoiesis [65], favor disease progression and decrease survival.

The main strengths of this study are (1) its multicenter design, (2) the development of predictive scores for three endpoints that are relevant for treatment planning in older patients with CRC, (3) the scores are based on factors that can be easily obtained in daily practice, and 4) the scores are simple and easy to use. There are also some limitations: (1) although the bootstrap methodology was used for internal validation, there is no external validation; thus, we do not know whether the score could be used in a different population. (2) Patients were receiving chemotherapy; thus, the validity of the scores in patients receiving targeted therapy or immunotherapy remains unknown. (3) Other endpoints may be relevant in the geriatric population, such as functional decline and the appearance of grade 2 toxicities. (4) A few patients required assistance to complete the geriatric assessment, which could have affected the validity of some items in their evaluation. (5) Our participants are not representative of the whole population of older patients with CRC, because they have been considered suitable for chemotherapy after a geriatric assessment. This excludes those patients with comorbid conditions precluding the use of such therapy.

## 5. Conclusions

Cancer treatment in older patients remains a challenge. The present study showed that a combination of geriatric, tumor and laboratory variables can predict the risk of severe toxicity, UH and ED. These tools can be used to plan treatment in older patients and implement measures aimed at reducing complications. Single agent chemotherapy or low dose combinations should be selected for patients at risk. Corrective measures should be initiated to improve the global health status, with special attention to factors most related to complications, i.e., deterioration of functional status (physical therapy, exercise, occupational therapy…) and poor nutritional status (nutrition consult, dietary recommendations, supplements…). These measures should start before the initiation of chemotherapy and should be kept over time, with frequent patient reevaluation.

## Figures and Tables

**Table 1:** Patient characteristics.

Characteristic	Total (*n* = 215)
Age, median (SD)	78 (4.9)
Sex	
Male	125 (58%)
Female	90 (32%)
Metastatic status	
M0	110 (51%)
M1	105 (49%)
Chemotherapy	
Standard therapy	100 (47%)
Reduced therapy or monotherapy	115 (53%)
Capecitabine	77 (36%)
Capecitabine-Oxaliplatin	33 (15%)
Oxaliplatin-5FU-anti-EGFR	32 (15%)
Oxalipatin-5FU-Bevacizumab	40 (18%)
Oxaliplatin-Irinotecan-5FU	15 (7%)
Irinotecan-anti-EGFR	6 (3%)
Capecitabine-Bevacizumab	12 (6%)
MAX2 index	
0	73 (34%)
1	127 (59%)
2	15 (7%)
ECOG PS	
0	58 (27%)
1	144 (67%)
2	13 (6%)
IADL	
8	97 (45%)
≤7	118 (55%)
ADL	
6	181 (84%)
≤5	34 (16%)
Number of falls in the past 6 months	
None	183 (85%)
≥1	32 (15%)
SPPB	
>7	185 (86%)
≤6	30 (14%)
Charlson comorbidity score	
0	80 (37%)
1	64 (30%)
≥2	71 (33%)
Pfeiffer test	
0–2 errors	185 (86%)
≥3 errors	30 (14%)
Unintentional weight loss %	
≤10%	181 (88%)
>10%	34 (12%)
VES 13	
0–2	103 (48%)
≥3	119 (52%)
Toxicity	
G3-5	73 (34%)
G0-2	142 (66%)
Early death < 6 months	
Yes	47 (22%)
No	168 (78%)
Unplanned hospitalizations	
Yes	60 (28%)
No	155 (67%)

Abbreviations: SD: Standard deviation, 5FU: 5-Fluorouracil, EGFR: Epidermal Growth Factor Receptor, ADL: Activity of Daily Living, IADL: Instrumental activity of Daily Living, ECOG PS: Eastern Cooperative Oncology Group performance status. SPPB, Short Physical Performance Battery, VES-13: Vulnerable Elders Survey-13.

**Table 2:** Factors associated with toxicity and early death and unplanned hospitalizations.

Variable	Toxicity G3–4			Early Death			Unplanned Hospitalizations		
	No	Yes	*p* Value	No	Yes	*p* Value	No	Yes	*p* Value
ECOG PS			0.95			0.91			**0.031**
2	9	4		10	3		6	7	
0–1	133	69		158	44		149	53	
IADL			**0.04**	9672		0.20			**0.0001**
≤7	85	33			22		98	20	
8	57	40			25		57	40	
ADL			0.85			**0.038**			**0.004**
≤5	22	12		22	12		16	18	
6	120	61		146	35		139	42	
Charlson comorbidity score			0.56			**0.008**			**0.015**
≥2	45	26		48	23		58	12	
0–1	97	47		120	26		96	482	
Unintentional weight loss %			**0.012**			**0.000**			**0.0001**
>5%	40	33		40	33		37	36	
≤5%	102	40		128	14		118	24	
Creatinine Clearance mL/min			**0.022**			0.26			**0.0001**
<50	42	33		60	21		36	46	
≥50	100	40		108	26		119	14	
Albumin g/dL			0.17			**0.000**			0.51
≤35	24	18		23	19		32	10	
>35	118	55		145	28		123	50	
Hemoglobin (g/dL)			0.61			**0.02**			0.48
<11	27	16		28	15		32	15	
≥11	1157	57		140	32		123	45	
Metastatic status			0.49			**0.000**			**0.000**
M1	67	38		69	36		64	41	
M0	75	35		99	11		91	19	
Chemotherapy			0.37			0.96			0.37
Standard therapy	63	37		78	22		75	25	
Reduced/monotherapy	79	36		90	25		80	35	
MAX2 index			**0.03**			0.73			0.84
≥0.45	87	55		110	32		103	39	
0–0.44	55	18		58	15		52	21	

Abbreviations: ECOG PS: Eastern Cooperative Oncology Group performance status, ADL: Activity of Daily Living, IADL: Instrumental activity of Daily Living.

**Table 3:** Variables significantly associated with toxicity grade 3–5.

Variable	β	SE	*p* †	HR (95% CI)	Score
MAX2 index > 0.45	0.796	0.315	0.009	2.176 (1.143–4.213)	2
Weight loss > 5%	0.709	0.315	0.03	2.014 (1.084–3.969)	2
IADL ≤ 7	0.455	0.284	0.04	1.293 (1.013–2.318)	1
Creatinine Clearance < 50 mL/min	0.628	0.299	0.03	1.891 (1.061–3.384)	1

SE: Standard error, CI: confidence interval, HR: hazard ratio, *p* † values were calculated using a two-sided Wald test for multivariable analyses. IADL: Instrumental activity of Daily Living.

**Table 4:** Variables significantly associated with unplanned hospitalization.

Variable	β	SE	*p* †	HR (95% CI)	Score
Stage IV	0.997	0.312	0.001	2.732 (1.438–4.957)	2
Weight loss > 5%	0.824	0.392	0.029	2.241 (1.067–4.921)	2
Albumin ≤ 3.5 g/dL	0.739	0.399	0.045	2.012 (1.006–4.45)	2
Creatinine Clearance < 50 mL/min	0.878	0.3719	0.013	2.219 (1.149–4.352)	2
Charlson score ≥ 2	0.215	0.181	0.045	1.239 (1.001–1.573)	1

SE: Standard error, CI: confidence interval, HR: hazard ratio, *p* † values were calculated using a two-sided Wald test for multivariable analyses.

**Table 5:** Variables significantly associated with early death.

Variable	β	SE	*p* †	HR (95% CI)	Score
Stage IV	1.497	0.364	0.000	5.026 (10.109–2.486)	5
Weight loss > 5%	0.9438	0.369	0.009	2.542 (1.243–5.106)	2
Hemoglobin ≤ 11 g/dL	0.823	0.354	0.019	2.213 (1.1352–4.518)	2
ADL ≤ 5	0.437	0.311	0.045	1.104 (1.004–2.369)	1

SE: Standard error, CI: confidence interval, HR: hazard ratio, *p* † values were calculated using a two-sided Wald test for multivariable analyses.

## Data Availability

De-identified individual data might be made available following publication by reasonable request to the corresponding author.

## Supplementary Materials

The following are available online at https://www.mdpi.com/article/10.3390/cancers14010127/s1, Figure S1: Receiver operating characteristic (ROC) analyses to assess the capacity of the predicting grade 3–4 toxicity, Table S1: Summary of CGA Domains and Elements.
