# Supplementary material for: Prediction of Chemotoxicity, Unplanned Hospitalizations and Early Death in Older Patients with Colorectal Cancer Treated with Chemotherapy
Source: Cancers (Basel). 2021 Dec 28;14(1):127. doi: 10.3390/cancers14010127 (PMC8749992; doi:10.3390/cancers14010127)
Supplement: Supplementary file 1 [file cancers-14-00127-s001.zip › cancers-1528207-supplementary.pdf]

Supplementary Table S1. Summary of CGA Domains and Elements

Domains	Elements of Assessment	Range	Cut-off
Functional status	ECOG performance status	0-2	2
	Activities of daily living (26)	0-6	≤ 5
	Instrumental activities of daily living (27)	0-8	≤ 7
	Physical performance test SPPB (23)	0-12	≤ 6
	Nº of falls in the last 6 months		≥ 1
Comorbidity	Cumulative Illness Rating Scale for Geriatrics (CIRS-G score) (21)	0-56	≥ 14
	Charlson index (22)	0-25	≥ 2
Psycological status	Hospital Anxiety Scale (28)	0-21	≥ 11
	Hospital Depression Scale (28)	0-21	≥ 11
Cognitive status	Pfeiffer test (25)	0-3 vs ≥ 2	
Social support	MOS Social Support Survey (29)	0-25	>15
Nutritional status	Body mass index	-	<21
	Percent unintentional weight lost in the last 6 months (%)	-	≥5

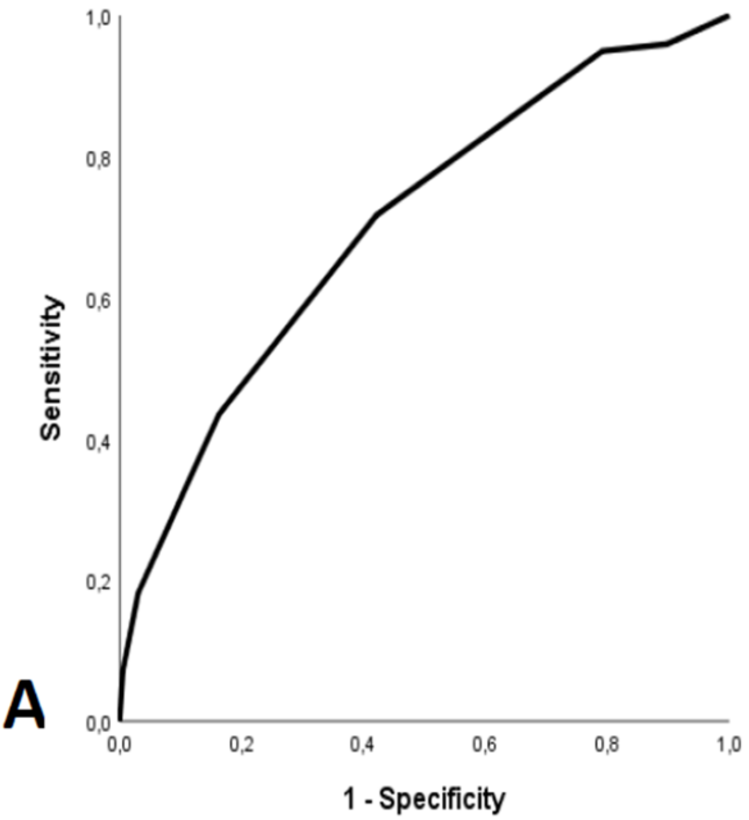

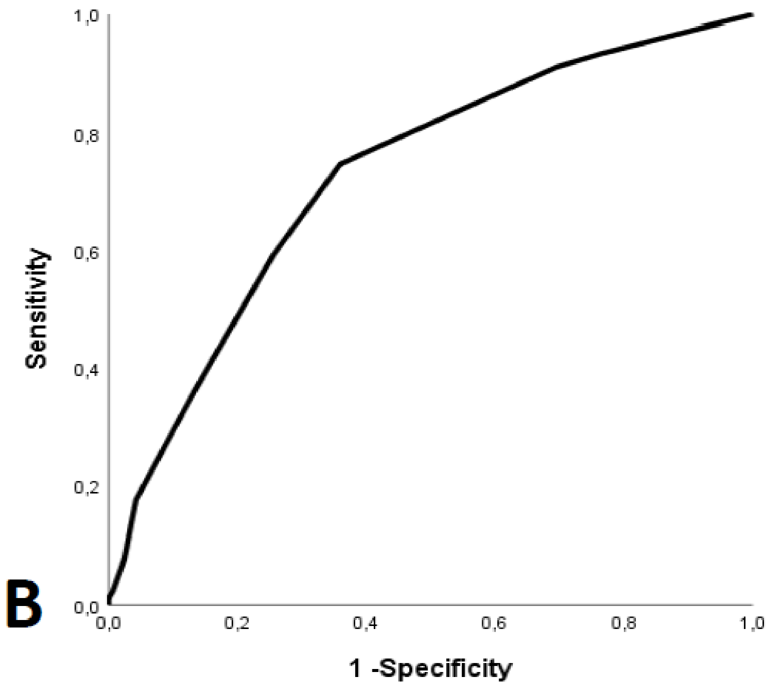

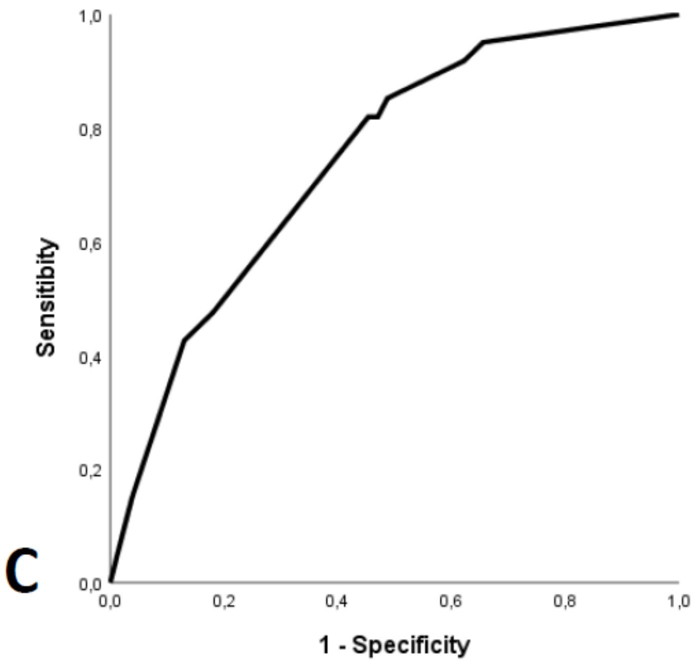

Figure S1. Receiver operating characteristic (ROC) analyses to assess the capacity of the predicting grade 3-4 toxicity (A), unplanned hospitalizations (B) and death at 6 months (C) .
